# Acylglycerol Kinase 2-mediated Inhibition of Sirtuin 2 Restores AMPK/AKT/mTOR Signaling Balance in Podocytes: A Pharmacological Strategy for Diabetic Nephropathy

**DOI:** 10.5812/ijpr-165603

**Published:** 2026-02-12

**Authors:** Zahra Zahid Piracha, Umar Saeed, Syed Muhammad Aun Raza, Zia Ashraf, Bazaid Muhammad, Mehedi Tubaishi Rubab, Aqsa Talib, Roshan Nadeem, Syed Shayan Gilani, Ayesha Yar Khan, Muhammad Usama Khan, Dilber Uzun Ozsahin, Andromeda M. Nauli, Surya M. Nauli

**Affiliations:** 1 Faculty of Rehabilitation and Allied Health Sciences, Riphah International University, Islamabad, Pakistan; 2 Szechenyi Istvan University, Gyor, Hungary; 3 Korea University College of Health Sciences, Korea University, Seongbuk-gu, Seoul, South Korea; 4 Clinical and Biomedical Research Center (CBRC), Foundation University School of Health Sciences (FUSH), Foundation University Islamabad (FUI), Islamabad, Pakistan; 5 Operational Research Center in Healthcare, Near East University, Nicosia, Turkey; 6 Department of Biomedical and Pharmaceutical Sciences, Chapman University, Irvine, United States; 7 International Center of Medical Sciences Research, Islamabad, Pakistan; 8 Akhtar Saeed Medical and Dental College, Lahore, Pakistan; 9 College of Allied Health Professionals, Government College University, Faisalabad, Pakistan; 10 University of Dhaka, Dhaka, Bangladesh; 11 Foundation University Islamabad, Islamabad, Pakistan; 12 Pak Red Crescent Medical College, Lahore, Pakistan; 13 Central Park Medical College, Lahore, Pakistan; 14 Department of Medical Diagnostic Imaging, College of Health Sciences, University of Sharjah, Sharjah, United Arab Emirates; 15 Department of Biomedical Sciences, School of Medicine, Western Michigan University Homer Stryker M.D., Kalamazoo, USA

**Keywords:** Intracellular Signaling, Energy Sensing, Histone Deacetylase, Diabetes, Kidney Injury, Drug Target

## Abstract

**Background:**

Diabetic nephropathy is a major cause of end-stage renal disease, driven in part by molecular dysfunctions in podocytes. Sirtuin 2 (Sirt2), a cytoplasmic NAD⁺-dependent deacetylase, has emerged as a potential regulator of key metabolic pathways, but its specific role in podocyte biology remains poorly defined.

**Objectives:**

This study aimed to investigate the function of Sirt2 in human podocytes (hPodo), delineate its interaction with histone deacetylase 6 (HDAC6), and evaluate the therapeutic potential of Sirt2 inhibition in restoring metabolic balance and protecting against diabetic nephropathy-associated podocyte stress.

**Methods:**

Comparative expression analysis was performed between hPodo and HEK293T kidney cells. Pharmacological inhibition of Sirt2 was carried out using acylglycerol kinase 2 (AGK2), alongside siRNA-mediated Sirt2 knockdown. AMPK/AKT/mTOR signaling activity was assessed by Western blotting and functional assays to determine metabolic and growth responses.

**Results:**

Human podocytes exhibited significantly elevated Sirt2 expression and high levels of HDAC6, forming a unique Sirt2–HDAC6 regulatory complex. Inhibition or silencing of Sirt2 induced robust AMPK activation while suppressing AKT/mTOR signaling. This signaling reprogramming restored energy sensing and attenuated hyperactive growth pathways, alleviating podocyte stress. Acylglycerol kinase 2 treatment reestablished metabolic homeostasis by disrupting Sirt2-mediated repression of AMPK.

**Conclusions:**

Sirtuin 2 inhibition, particularly through AGK2, emerges as a novel pharmacological strategy to protect podocytes, restore metabolic regulation, and potentially slow the progression of diabetic nephropathy. Significance Statement By inhibiting one of the important intracellular signaling pathways in human kidney cells, we could reduce the cellular stress that is commonly observed in diabetic kidney injury. This could serve as a drug target to slow the progression of kidney disease associated with diabetes mellitus.

## 1. Background

Acylglycerol kinase 2 (AGK2), also known as (2S)-2-[[(4-methylphenyl)sulfonyl]amino]-N-(1-phenylethyl)-3-phenylpropanamide, is a selective and cell-permeable inhibitor of Sirtuin 2 (Sirt2). Acylglycerol kinase 2 has emerged as a promising pharmacological agent for modulating cellular metabolism, inflammation, and stress responses in chronic diseases. Its therapeutic relevance is increasingly recognized in conditions where Sirt2 dysregulation contributes to pathogenesis, including diabetic nephropathy. Despite mounting evidence implicating Sirt2 in renal injury, its precise role in diabetic nephropathy remains incompletely understood, and AGK2 has not been extensively explored as a targeted pharmacological intervention in this context. Diabetic nephropathy is a debilitating microvascular complication of diabetes mellitus and remains a major cause of end-stage renal disease worldwide (1). Characterized by progressive glomerulosclerosis, podocyte loss, and chronic inflammation, diabetic nephropathy places a significant burden on affected individuals and healthcare systems (2). While current treatments emphasize glycemic control and blood pressure regulation, these approaches do not adequately target the molecular mechanisms driving renal damage, highlighting the urgent need for mechanism-based therapies. Sirt2, a member of the sirtuin family of NAD⁺-dependent deacetylases, has been identified as a key regulator of cellular processes relevant to diabetic nephropathy, including oxidative stress, inflammation, and energy metabolism (3-5). Its dysregulation has been implicated in a broad range of diseases such as cancer, neurodegenerative disorders, and metabolic syndromes (6-8). However, its role in kidney pathology is still debated, with some studies suggesting beneficial effects while others report context-dependent or even deleterious outcomes (9, 10). In cardiovascular systems, Sirt2 has shown protective roles (11, 12), but in renal cells, it appears to modulate insulin signaling and glucose uptake via AKT activation in podocytes (13). Additionally, its expression correlates with that of podocin, a podocyte-specific protein critical for maintaining glomerular filtration barrier integrity, suggesting a potential role in podocyte survival (14). This dualistic nature of Sirt2 function, both protective and pathogenic, adds complexity to its therapeutic targeting in diabetic nephropathy. The AMPK/AKT/mTOR pathway plays a pivotal role in diabetic nephropathy pathogenesis by regulating energy sensing, cell survival, and growth responses (15). Dysregulation of this pathway in podocytes and mesangial cells leads to cellular hypertrophy, fibrosis, and glomerulosclerosis, which are hallmark features of diabetic nephropathy (16-19). In particular, reduced AMPK activity alongside hyperactive AKT/mTOR signaling contributes to metabolic imbalance, making this cascade a critical target for pharmacological modulation. Histone deacetylase 6 (HDAC6) has also been identified as a modulator of renal injury. Inhibition of HDAC6 has shown renoprotective effects in diabetic nephropathy models by attenuating oxidative stress, fibrosis, and inflammation (20). As a primarily cytoplasmic deacetylase, HDAC6 influences protein trafficking, cytoskeletal dynamics, and stress granule formation (21). Despite these findings, the functional relationship between Sirt2 and HDAC6 in podocytes remains poorly defined. In this study, we investigate the pharmacological effects of AGK2, a selective Sirt2 inhibitor, on AMPK/AKT/mTOR signaling in human podocytes (hPodo) and HEK293T cells. Human podocytes were selected for their physiological relevance to glomerular function, as dysregulation of the AMPK/AKT/mTOR pathway in these cells is critically implicated in renal pathologies. HEK293T cells were employed as a complementary model due to their well-characterized signaling background, high transfectability, and reproducibility in pharmacological studies, thereby enabling detailed mechanistic evaluation of AGK2-mediated Sirt2 inhibition. By assessing the impact of Sirt2 inhibition on AMPK activation and downstream growth pathways, and examining its interaction with HDAC6, we aim to elucidate the molecular mechanisms underlying Sirt2-mediated regulation of diabetic kidney injury. 

## 2. Objectives

Our findings highlight the therapeutic potential of AGK2 in rebalancing metabolic signaling in diabetic nephropathy and support Sirt2 as a pharmacological target for preventing podocyte dysfunction and disease progression.

## 3. Methods

### 3.1. Cell Culture

Human podocytes and HEK293T cells were cultured in Dulbecco's Modified Eagle Medium (DMEM) supplemented with 1% penicillin-streptomycin and 10% fetal bovine serum (Gibco BRL) under controlled conditions (37°C, 5% CO₂). HEK293T cells underwent passaging every other day, while hPodo cells were passaged every third day. Additionally, hPodo cells were cultured as mentioned above, with the inclusion of 1X podocyte growth supplements (insulin-transferrin-selenium; Thermo Fisher #41400045) (22).

### 3.2. Stable Cell Lines

shControl and shSIRT2-#1–2 cells were generated as described previously (23).

### 3.3. SDS-PAGE and Western Blotting

Equal amounts of cell lysate, as previously detailed (23, 24), were prepared and subjected to 12% SDS-PAGE gels. The resolved proteins were subsequently transferred onto PVDF membranes and incubated with the appropriate primary antibodies at a dilution of 1:1000 (refer to Table 1). This was followed by incubation with anti-rabbit or anti-mouse secondary antibodies, both coupled to horseradish peroxidase, at a dilution of 1:5,000 (Thermo Fisher Scientific). The blots were visualized using enhanced chemiluminescence (Amersham), and the relative intensities of the bands were quantified using ImageJ 1.46r.

**Table 1.** Antibodies Used in the Study for Western Blotting

Antibody Target	Species	Experiment	Supplier	Catalog No.
**Sirt1 **	Mouse monoclonal		Abcam	Ab110304
**Sirt2 **	Rabbit polyclonal	SDS-PAGE-Immunoblotting (IB)/ Immunoprecipitation (IP)	Santa Cruz	sc-20966
**Sirt3**	Rabbit monoclonal	SDS-PAGE-IB	Cell Signaling Technology	#5490
**Sirt4**	Rabbit polyclonal	SDS-PAGE-IB	Cell Signaling Technology	#69786
**Sir5**	Rabbit monoclonal	SDS-PAGE-IB	Cell Signaling Technology	#8782
**Sirt6**	Rabbit monoclonal	SDS-PAGE-IB	Cell Signaling Technology	#12486
**Sirt7**	Rabbit monoclonal	SDS-PAGE-IB	Cell Signaling Technology	#5360
**HDAC6**	Mouse monoclonal	SDS-PAGE-IB/IP	Santa Cruz	sc-28386
**GAPDH **	Mouse monoclonal	SDS-PAGE-IB	Santa Cruz	sc32233
**p-AMPK (T172) **	Rabbit polyclonal	SDS-PAGE-IB	Cell Signaling Technology	#2535
**AMPK **	Rabbit polyclonal	SDS-PAGE-IB	Cell Signaling Technology	#2532
**p-AKT (S437) **	Rabbit monoclonal	SDS-PAGE-IB	Cell Signaling Technology	#9271
**p-AKT (T308) **	Rabbit polyclonal	SDS-PAGE-IB	Cell Signaling Technology	#9275
**AKT **	Rabbit polyclonal	SDS-PAGE-IB	Cell Signaling Technology	#9272
**mTOR **	Rabbit polyclonal	SDS-PAGE-IB	Cell Signaling Technology	#2972

### 3.4. Acylglycerol kinase 2 Treatment

To examine the effects of a Sirt2 inhibitor on the AMPK/AKT/mTOR pathway in hPodo, AGK2 (Sigma-Aldrich #A8231) 8 was dissolved in dimethyl sulfoxide (DMSO). In brief, HEK293T cells or hPodo cells were seeded onto 6 cm plates. Cells were exposed to 10 µM AGK2 24 hours post seeding. Lysates were prepared at 72 hours post-treatment.

### 3.5. Cell Cytotoxicity Assay

The cytotoxic effects of AGK2 were assessed in both HEK293T cells and hPodo cell lines. Cells were seeded in 96-well microplates and treated with varying concentrations of AGK2 for 48 hours at 37°C. The 3-(4,5-dimethylthiazol-2-yl)-2,5-diphenyltetrazolium bromide (MTT) assay was performed as described previously (25, 26). Subsequently, the concentrations of AGK2 at which cell viability was reduced to 50% (CC50) compared to the control were determined.

### 3.6. Co-immunoprecipitation

To investigate the physical interaction between Sirt2 and HDAC6, cell lysates from both HEK293T and hPodo were subjected to immunoprecipitation using mouse monoclonal anti-HDAC6 antibodies. Subsequently, the immunoprecipitated samples were immunoblotted with rabbit polyclonal anti-Sirt2 antibodies. As a negative control for immunoprecipitation, mouse normal IgG (Merck Millipore #12-371) was utilized. The lysates were separated by SDS-PAGE on 10% gels and transferred onto PVDF membranes for immunoblotting with primary antibodies including anti-HDAC6, anti-GAPDH, and anti-Sirt2. This was followed by incubation with anti-mouse or anti-rabbit secondary antibodies conjugated to horseradish peroxidase. Immunoblots were visualized using enhanced chemiluminescence.

### 3.7. RNA Interference

Sirt2 siRNA was purchased from Santa Cruz #sc-40989. Human podocytes cells were transfected with siRNA using Lipofectamine 2000 according to the manufacturer’s instructions. At 24 hours after transfection, the cells were used for further experiments.

### 3.8. Statistical Analysis

The data were presented as the mean ± standard deviation. Mean values were compared utilizing Student’s *t*-test. P-values less than 0.05 were regarded as statistically significant.

## 4. Results

### 4.1. Comparative analysis of Sirtuin expression levels in HEK293T cells and hPodo reveals differential regulation of Sirt2

**Figure 1.**
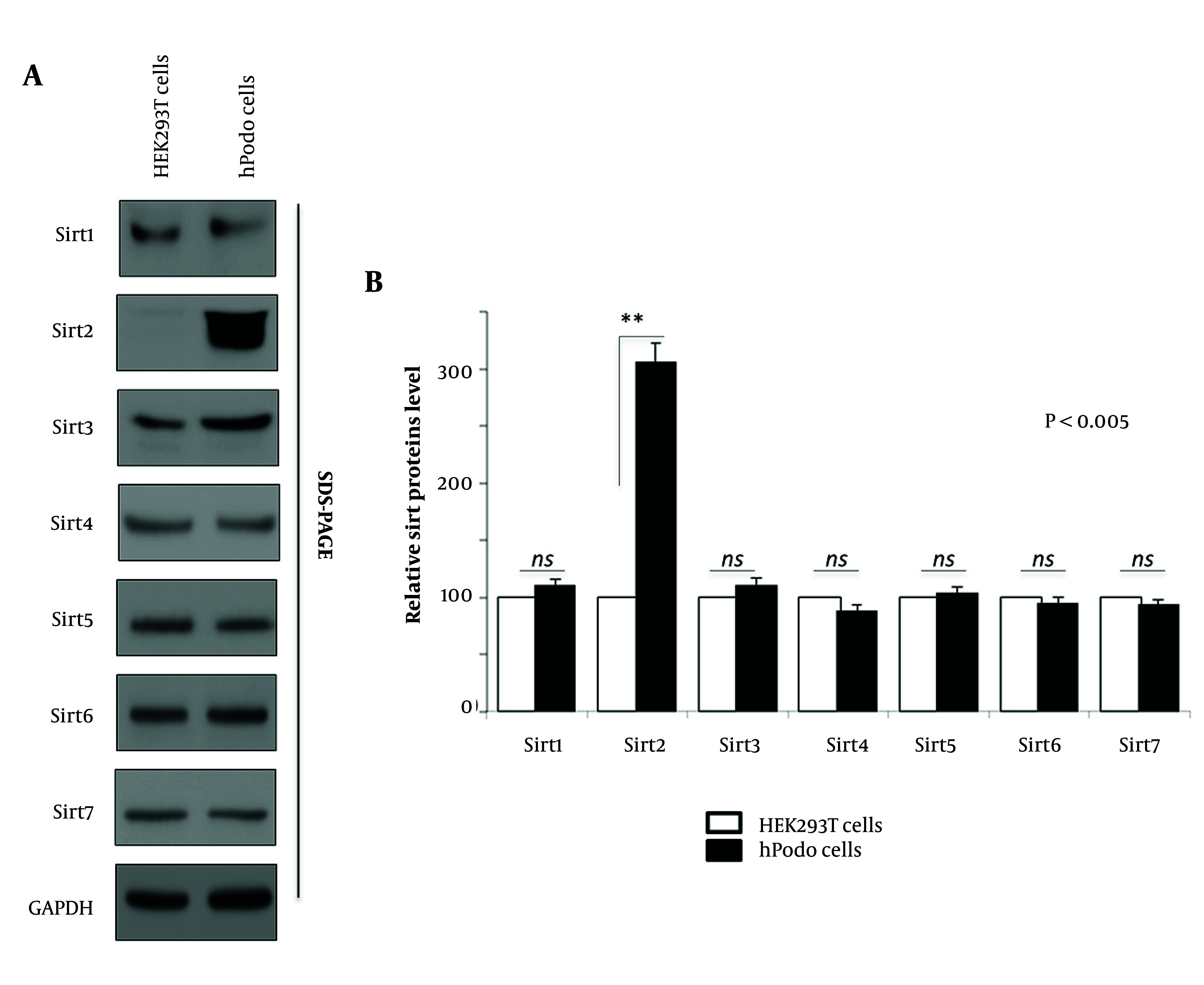
Sirt2 is upregulated in Human podocytes (hPodo) cells. A, newly plated HEK293T and hPodo cells (lanes 1 and 2) were cultured for 72 hours, and lysates were prepared thereafter. Specific antibodies (Sirtuin 1 to 7) were utilized for protein visualization, with GAPDH serving as the loading control; B, relative Sirtuin protein levels are depicted, representing the mean relative levels from three independent experiments. Statistical significance was determined using Student’s *t*-test, with exact P-values relative to the respective control displayed.

Sirtuins, a class of NAD⁺-dependent histone deacetylases, are critical regulators of cellular metabolism, stress response, and longevity (4, 5). While several studies have elucidated their roles in various cellular processes, the differential regulation of Sirtuin expression across different cell types remains an area of active investigation. Sirtuins, particularly Sirt2, have emerged as potential regulators of cellular pathways implicated in diabetic nephropathy pathogenesis (3). Here, we investigated the expression levels of Sirt1 to Sirt7 in HEK293T cells and hPodo cells (Figure 1A) to discern potential differences in their regulation. Remarkably, among the examined Sirtuins, Sirt2 exhibited a significant increase in hPodo cells compared to HEK293T cells (Figure 1 lane 1 vs. 2). Whereas the expression levels of other Sirtuins remained relatively consistent between the two cell lines (Figure 1 lane 1 vs. 2). Figure 1B illustrates the graphical depiction of data obtained from three independent experiments, showcasing the comparative levels of Sirtuins between HEK293T cells and hPodo cells. Only Sirt2 shows a significant increase in the levels (P < 0.005). Notably, this observation contrasts with the expression patterns of other Sirtuins examined in this study, suggesting a unique regulatory mechanism governing Sirt2 expression in hPodo cells. This finding underscores the importance of considering cell-type-specific differences in Sirtuin regulation and highlights Sirt2 as a potential key player in human podocyte cell physiology and associated pathologies, such as diabetic nephropathy.

### 4.2. Elevated Expression of HDAC6 in hPodo Cells and its Interaction with Sirt2

**Figure 2.**
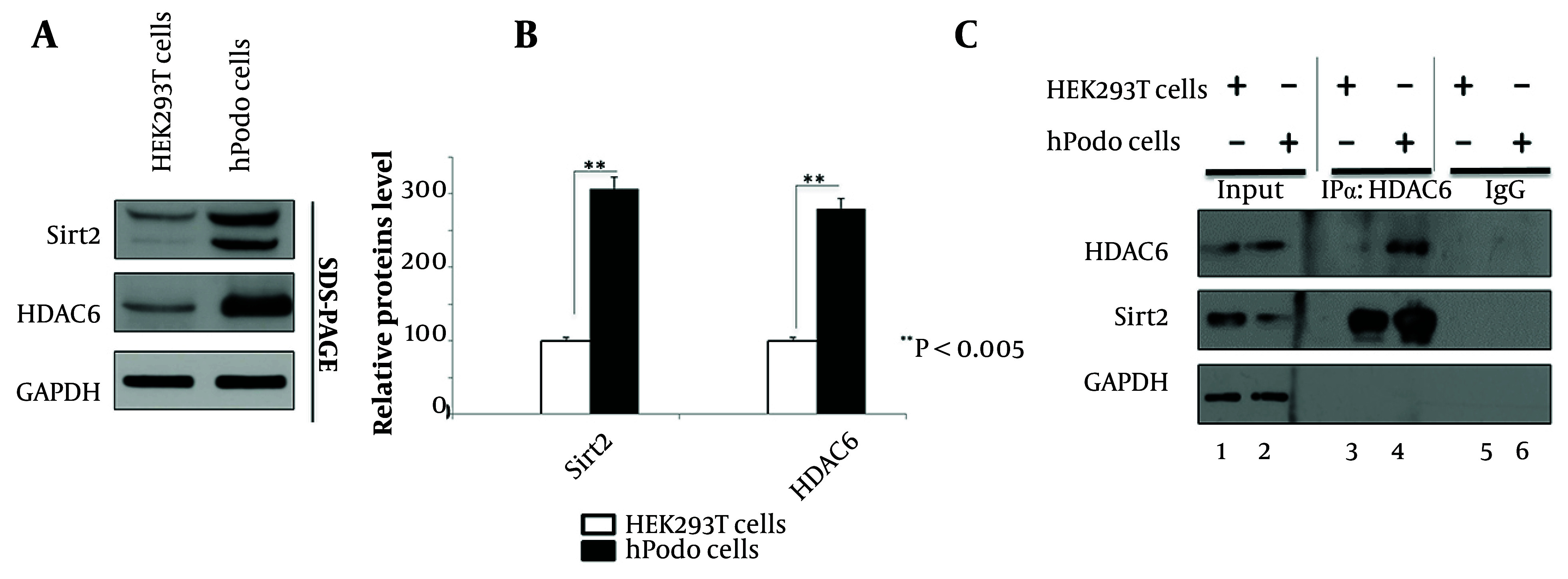
HDAC6 and Sirt2 interact in human podocytes (hPodo) cells. A, HEK293T and hPodo cells, newly plated (lanes 1 and 2), were cultured for 72 hours, followed by lysate preparation. Protein visualization was performed using HDAC6 and Sirt2 antibodies, with GAPDH employed as a loading control; B, relative protein levels of Sirt2 and HDAC6 are depicted as the mean relative levels from three independent experiments. Statistical significance was assessed using Student’s *t*-test, and exact P-values relative to the respective control are provided; C, immunoprecipitation was conducted on HEK293T cells or hPodo cells using HDAC6 antibody and subsequently immunoblotted with Sirt2 antibody, three days post-seeding. Rabbit normal IgG was utilized as a negative control.

Recent studies have implicated HDAC6 dysregulation in diabetic nephropathy pathogenesis, including alterations in cytoskeletal dynamics and inflammatory responses (20, 21). Additionally, elevated Sirt2 expression has been observed in podocytes (Figure 1). Understanding the interplay between Sirt2 and HDAC6 in podocytes may provide crucial insights into diabetic nephropathy mechanisms. Thus, we examined HDAC6 levels in HEK293T cells and hPodo cells (Figure 2A). Our data revealed upregulated HDAC6 expression in hPodo cells compared to HEK293T cells (Figure 2 lane 1 vs. 2). Figure 2B illustrates the comparative levels of Sirt2 and HDAC6 between HEK293T cells and hPodo cells, demonstrating a significant increase in both proteins (P < 0.005). Previous studies have reported the physical and functional interaction between Sirt2 and HDAC6 (11, 27, 28). We further investigated whether this interaction exists between Sirt2 and HDAC6 in hPodo cells (Figure 2C). Our data revealed that HDAC6 interacts with Sirt2, and this interaction is strengthened in hPodo cells compared to HEK293T cells (Figure 2 lane 3 vs. 4). These findings unveil an enhanced expression of HDAC6 in hPodo cells, accompanied by a reinforced interaction between Sirt2 and HDAC6. Thus, suggesting a potential regulatory role for the Sirt2–HDAC6 axis in podocyte biology and diabetic nephropathy progression. Further elucidation of the mechanistic implications of this interaction may uncover novel therapeutic avenues for diabetic nephropathy.

### 4.3. Silencing Sirt2 Results in the Activation of AMPK and Suppression of the AKT/mTOR Pathway 

**Figure 3.**
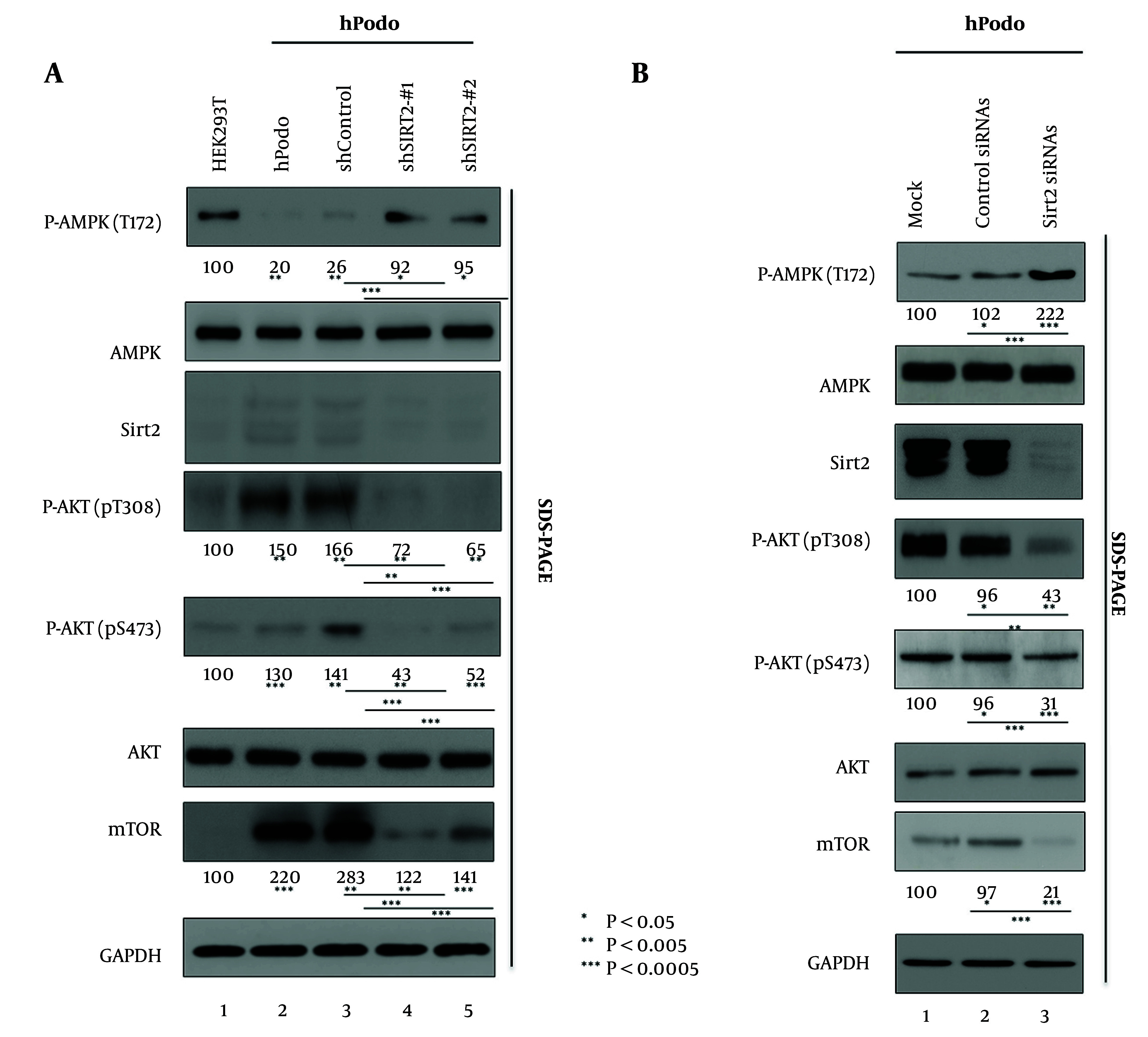
Knocking down of Sirt2 causes AMPK activation and AKT/mTOR supression. A, HEK293T cells (lane 1) and human podocytes (hPodo) cells (lanes 2 - 4) were transduced with lentivirus-like particles containing either control shRNA (shControl) (lane 1) or SIRT2-targeting shRNAs (shSIRT2-#1, shSIRT2-#2) (lanes 2 and 3). Cell lysates were obtained 72 hours post-transduction; B, in hPodo cells, siRNA treatment was performed, with cells treated with control siRNAs (lane 2) or Sirt2 siRNAs (lane 3). Cell lysates were obtained 72 hours post-seeding. The relative levels of total and active AMPK (T172), AKT (pT308 and pS473), and mTOR were quantified using ImageJ 1.46r. Data represent the mean values from three independent experiments. Statistical significance was assessed using Student’s *t*-test, with *P < 0.05, **P < 0.005, and ***P < 0.0005 (relative to respective controls) indicated.

The significance of AMP-activated protein kinase (AMPK) in diabetic nephropathy is underscored by its protective role against renal injury, achieved through the regulation of cellular metabolism, inflammation, and oxidative stress. Perturbations in AMPK signaling have been identified as a contributing factor in the development of diabetic nephropathy, thus presenting a potential avenue for therapeutic intervention (15). Additionally, the involvement of AKT signaling in diabetic nephropathy progression is noteworthy, particularly in its impact on podocyte function, inflammation, and fibrosis. Activation of AKT has been associated with renal hypertrophy and glomerular dysfunction in diabetes (17). Furthermore, the role of mechanistic target of rapamycin (mTOR) signaling in diabetic nephropathy is significant, as it contributes to renal hypertrophy, fibrosis, and inflammation. Dysregulated mTOR activity has been implicated in podocyte injury and albuminuria, highlighting its relevance as a potential therapeutic target in diabetic nephropathy (16). Previous studies have highlighted the regulatory role of Sirt2 in modulating the AMPK/AKT/mTOR pathway in cancer cells, demonstrating its regulatory effects on cellular metabolism and proliferation (29). Hence, we investigated this pathway in HEK293T cells and in hPodo cells (Figure 3 lane 1 vs. 2). Our analysis revealed distinct expression patterns within the AMPK/AKT/mTOR pathway in hPodo cells compared to HEK293T cells (Figure 3 lane 1 vs. 2). Specifically, we observed a downregulation of AMPK phosphorylated at T172 (AMPK pT172) in hPodo cells, accompanied by increased expression levels of AKT phosphorylated at T308 and S473, as well as mTOR (Figure 3 lane 1 vs. 2). To elucidate the potential regulatory role of Sirt2 in this pathway, we investigated the consequences of Sirt2 knockdown in hPodo cells (Figure 3 lane 3 vs. 4 and 5). Remarkably, upon Sirt2 knockdown, we observed a reversal of expression patterns within the AMPK/AKT/mTOR pathway. Specifically, AMPK pT172 expression increased, while the expression levels of AKT phosphorylated at T308 and S473, as well as mTOR, decreased compared to shControl cells. Next, in the subsequent experiment, we investigated the impact of siRNA-mediated knockdown of Sirt2 on the AMPK/AKT/mTOR pathway in hPodo cells (Figure 3B). Following treatment with siRNA targeting Sirt2, we observed significant alterations in the expression levels within the AMPK/AKT/mTOR pathway in hPodo cells. Specifically, the expression of AMPK phosphorylated at T172 (AMPK pT172) was notably elevated compared to shControl cells, indicating increased activation of AMPK signaling upon Sirt2 knockdown (Figure 3 lane 2 vs. 3). Conversely, the expression levels of AKT phosphorylated at T308 and S473, as well as mTOR, were decreased in hPodo cells treated with siRNA targeting Sirt2 compared to shControl cells (Figure 3 lane 2 vs. 3). These findings suggest that knockdown of Sirt2 leads to a shift in the activation status of the AMPK/AKT/mTOR pathway in hPodo cells. The observed increase in AMPK activation coupled with decreased AKT and mTOR phosphorylation levels upon Sirt2 knockdown highlights the potential regulatory role of Sirt2 in modulating this signaling pathway in podocytes. Further investigation into the molecular mechanisms underlying these effects is warranted to fully elucidate the role of Sirt2 in podocyte biology and its implications for diabetic nephropathy pathogenesis.

### 4.4. Acylglycerol Kinase 2 Treatment Leads to the Activation of AMPK and Suppression of the AKT/mTOR Pathway

**Figure 4.**
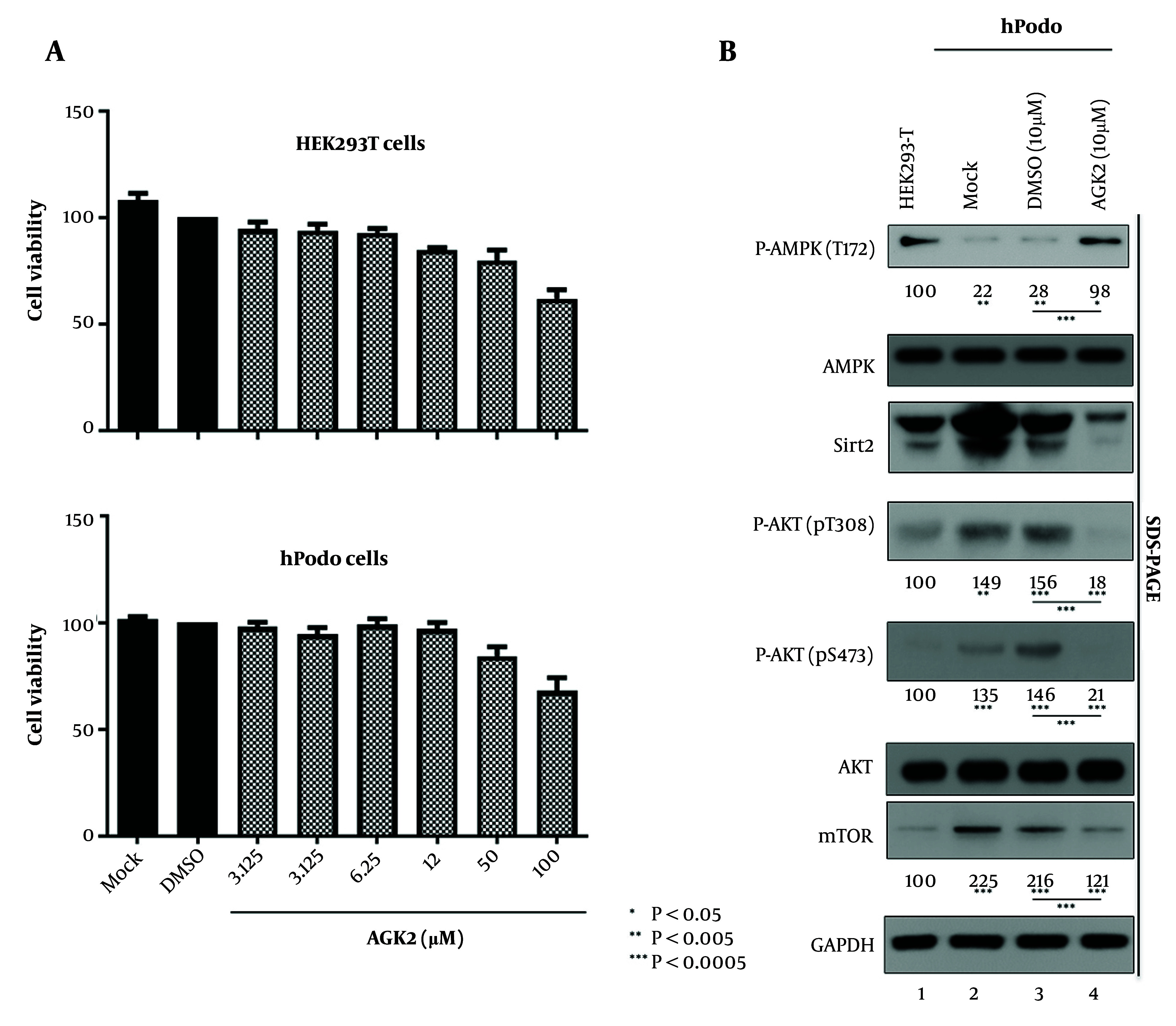
Acylglycerol kinase 2 (AGK2) treatment causes AMPK activation and ATK/mTOR suppression. A, An MTT assay was conducted to assess the cytotoxicity of the Sirt2 inhibitor AGK2; B, HEK293T cells (lane 1) and human podocytes (hPodo) cells (lanes 2 - 4) were freshly seeded. Lane 3 was treated with DMSO (10 µM), while lane 4 was treated with AGK2 (10 µM). Lysates were prepared 72 hours post-seeding, and the relative levels of total and active AMPK (T172), AKT (pT308 and pS473), and mTOR were quantified using ImageJ 1.46r. Data represent the mean values from three independent experiments. Statistical significance was determined using Student’s *t*-test, with * P < 0.05, **P < 0.005, and *** P < 0.0005 (relative to respective controls) indicated.

The therapeutic promise of histone deacetylase (HDAC) inhibitors, notably HDAC6 inhibitors, in the management of renal and cardiac ailments is reported. This is elucidated through preclinical investigations showcasing the effectiveness of HDAC inhibitors in mitigating kidney injury, fibrosis, and inflammation, suggesting their potential applicability in treating diabetic nephropathy (19). In the subsequent experiment, we evaluated the effects of AGK2, a Sirt2 inhibitor, on the AMPK/AKT/mTOR pathway in hPodo cells (Figure 4), considering the potential implications for diabetic nephropathy treatment. We conducted an MTT assay to ascertain the appropriate concentration of AGK2, which minimally affects cell viability while effectively inhibiting Sirt2 activity. Various concentrations of AGK2 were tested to establish a suitable dose range for subsequent experiments in HEK293T cells and in hPodo cells (Figure 4A). The concentration of 10 µM AGK2 was selected as the optimal concentration for further experiments. Our data revealed notable alterations in the expression levels within the pathway. Specifically, we observed a significant increase in the expression of AMPK phosphorylated at T172 (AMPK pT172), indicative of enhanced AMPK activation, following AGK2 treatment (Figure 4 lane 3 vs. 4). In contrast, the expression levels of AKT phosphorylated at T308 and S473, as well as mTOR, were decreased in hPodo cells treated with Acylglycerol kinase 2 compared to control cells (Figure 4 lane 3 vs. 4). These findings suggest that inhibition of Sirt2 activity by AGK2 leads to a shift in the activation status of the AMPK/AKT/mTOR pathway in hPodo cells. The observed increase in AMPK activation and decreased phosphorylation levels of AKT and mTOR may have therapeutic implications for diabetic nephropathy treatment. Further investigation is warranted to elucidate the precise mechanisms underlying these effects and to assess the potential utility of AGK2 as a therapeutic intervention for diabetic nephropathy.

## 5. Discussion

The emergence of AGK2 as a pharmacological inhibitor of Sirt2 offers a promising therapeutic approach to mitigate podocyte injury and signaling imbalance in diabetic nephropathy. This study presents comprehensive mechanistic insights into how AGK2 modulates key intracellular signaling pathways, most notably the AMPK/AKT/mTOR cascade, through targeted inhibition of Sirt2 in human podocytes. These findings add to the growing body of evidence that Sirt2 is a critical regulatory hub in metabolic and stress-related signaling networks implicated in renal disease progression. The sirtuin family (SIRT1–SIRT7) comprises NAD⁺-dependent class III HDACs that control diverse biological processes, including aging, oxidative stress response, chromatin remodeling, and energy metabolism (30, 31). Among these, Sirt2 is primarily localized in the cytoplasm, where it regulates non-histone proteins involved in mitosis, cytoskeletal organization, and metabolic signaling. Our comparative expression analysis between HEK293T and hPodo cells revealed a striking, podocyte-specific upregulation of Sirt2, indicating that this isoform may play a more pronounced role in glomerular epithelial cell function and dysfunction. This cell-type specificity has profound therapeutic implications. In podocytes, which are terminally differentiated and highly specialized, the tight regulation of cytoskeletal integrity and energy balance is essential for maintaining glomerular filtration barrier function. Dysregulation of these processes in the diabetic milieu is a hallmark of diabetic nephropathy, and the elevation of Sirt2 suggests a maladaptive adaptation to chronic metabolic stress. This positions Sirt2 not only as a marker of cellular stress in diabetic nephropathy, but also as a modifiable driver of disease progression.

Compellingly, our findings also underscore a functional interaction between Sirt2 and HDAC6, another cytoplasmic deacetylase known for regulating tubulin acetylation, autophagy, and stress granule dynamics (20, 21). HDAC6 has been previously implicated in diabetic nephropathy through its role in promoting fibrosis, cytoskeletal disorganization, and inflammatory signaling. The Sirt2–HDAC6 complex has been described in other pathologies, including Alzheimer’s disease, Parkinson’s disease, and rheumatoid arthritis, where it modulates immune responses and protein degradation (8, 32). Our data demonstrate a marked enhancement of Sirt2–HDAC6 interaction in hPodo cells, suggesting that this axis may similarly contribute to inflammation and cytoskeletal disruption in diabetic nephropathy. By inhibiting Sirt2 with AGK2, this pathological interaction may be attenuated, leading to improved cellular resilience.

Sirt2 also exerts direct effects on AKT signaling, a central regulator of cell growth, survival, and metabolism. It has been shown to deacetylate and stabilize AKT, thus promoting its activation (13). In the diabetic kidney, hyperactivation of AKT and its downstream effector mTOR has been implicated in podocyte hypertrophy, glomerular basement membrane thickening, and proteinuria (33). mTOR forms two distinct complexes, mTORC1 and mTORC2, of which mTORC1 plays a pivotal role in promoting pathological cellular growth in diabetic nephropathy. Our study demonstrates that Sirt2 inhibition via AGK2 suppresses phosphorylation of AKT and mTOR, pointing to a potential mechanism by which AGK2 interrupts the pathogenic growth signaling axis in podocytes. In parallel, Sirt2 inhibition led to robust activation of AMPK, a master regulator of energy homeostasis. AMPK activation is known to exert protective effects in diabetic nephropathy by inhibiting inflammation, oxidative stress, and fibrotic signaling (34). The ability of AGK2 to reactivate this pathway underscores its dual capacity to suppress pathological AKT/mTOR signaling while restoring energy-sensing and antioxidant defenses in diabetic podocytes. These combined actions position AGK2 as a multi-modal pharmacologic agent capable of recalibrating aberrant signaling landscapes in diabetic nephropathy.

From a therapeutic development standpoint, these findings are significant. The existing management of diabetic nephropathy remains largely supportive, focusing on glycemic control, renin-angiotensin system blockade, and recently, sodium-glucose cotransporter 2 (SGLT2) inhibitors. However, none of these treatments directly target podocyte-specific molecular alterations. The identification of Sirt2 as a key pathogenic node, and AGK2 as a selective inhibitor, opens the door to a new class of disease-modifying agents that intervene upstream in the signaling hierarchy. Additionally, AGK2 offers advantages as a small-molecule inhibitor, including ease of delivery, known selectivity for Sirt2, and amenability to chemical optimization for clinical use.

Nevertheless, several questions remain that warrant further investigation. While our in vitro data establish the effectiveness of AGK2 in reversing Sirt2-mediated signaling abnormalities in human podocytes, in vivo validation in diabetic nephropathy animal models is essential to confirm these findings under physiological conditions. Furthermore, the pharmacokinetics, tissue distribution, and renal safety profile of AGK2 remain to be fully elucidated. Investigating the long-term effects of Sirt2 inhibition on podocyte viability and renal function will also be critical before transitioning to clinical development. Additionally, the context-dependent nature of Sirt2 function observed in various organ systems necessitates a cautious, tissue-targeted therapeutic approach. While inhibition may be beneficial in the kidney, it could produce undesirable effects in other Sirt2-expressing tissues if not carefully modulated. Thus, the future development of kidney-targeted Sirt2 inhibitors or AGK2 analogs may offer an optimal balance between efficacy and safety.

### 5.1. Conclusion

This study provides novel and comprehensive evidence that pharmacological inhibition of Sirt2 by AGK2 restores homeostatic signaling in podocytes, significantly impacting the AMPK/AKT/mTOR axis, a core pathway in diabetic nephropathy pathogenesis. The findings support Sirt2 as a central regulator of metabolic and inflammatory dysfunction in diabetic nephropathy and position AGK2 as a promising candidate for targeted therapeutic intervention. Further preclinical and translational studies are warranted to advance this strategy toward clinical application, with the goal of developing innovative, mechanism-based therapies to improve outcomes for patients with diabetic nephropathy.

## Data Availability

The authors declare that all the data supporting the findings of this study are contained within the paper.
